# Folic acid and sperm quality improvement: insights from snRNA sequencing and RNA splicing mechanisms

**DOI:** 10.3389/fnut.2025.1648307

**Published:** 2025-08-29

**Authors:** Qifu He, Mingrong Zhang, Sheng Xiao, Zijing Guo

**Affiliations:** ^1^Wuhou District People's Hospital, Chengdu, China; ^2^Wuhou District Health Hospital Woman and Children, Chengdu, China; ^3^College of Animal and Veterinary Sciences, Southwest Minzu University, Chengdu, China

**Keywords:** folic acid, sperm quality, snRNA sequencing, RNA splicing, male fertility

## Abstract

Folic acid, an essential micronutrient in male reproductive physiology, serves as a critical cofactor in one-carbon metabolism by facilitating nucleotide biosynthesis and epigenetic methylation processes fundamental to spermatogenesis. Its metabolic role is characterized by two pivotal biochemical transformations: the remethylation of homocysteine to methionine and the subsequent generation of S-adenosylmethionine. These reactions collectively sustain nucleic acid synthesis, preserve genomic integrity, and modulate transcriptional regulation in developing germ cells. Compromised folate status disrupts small nuclear RNA (snRNA) maturation and methylation patterns, resulting in impaired spliceosome complex formation and compromised pre-messenger RNA (pre-mRNA) splicing accuracy. Such molecular perturbations generate defective transcripts that ultimately undermine proteomic homeostasis during spermiogenesis. Preclinical evidence demonstrates that folate deficiency induces chromosomal segregation errors, mitotic spindle checkpoint dysfunction, and concurrent oxidative/endoplasmic reticulum stress pathways—all converging to manifest as teratozoospermia, diminished motility, and elevated sperm DNA fragmentation indices. Folic acid supplementation can improve snRNA and spliceosomal function, leading to improve semen parameters, particularly in individuals with polymorphisms in folate-metabolizing enzymes such as MTHFR. However, treatment efficacy exhibits dose-dependence, temporal dynamics, pharmacogenetic variation, and synergistic interactions with concurrent micronutrient administration, underscoring the imperative for personalized nutritional approaches. Emerging single-nucleus RNA sequencing technologies have elucidated intricate regulatory circuitry connecting folate-responsive snRNAs with mRNA processing, miRNA-mediated silencing, and long noncoding RNAs (lncRNAs)-mediated chromatin remodeling. These findings propose candidate molecular signatures for monitoring therapeutic response. Notwithstanding these advances, the mechanistic interplay between folate metabolism and snRNA processing machinery remains incompletely characterized, and evidence-based clinical protocols for infertility management remain undefined. Future research directions should encompass: (1) multi-omics integration (epigenomic-transcriptomic-proteomic); (2) pharmacogenomic-guided intervention trials; and (3) dynamic splicing efficiency quantification platforms. Such approaches will enable precision therapeutic stratification to maximize clinical outcomes while mitigating potential adverse effects. This critical synthesis delineates the mechanistic nexus between folate-dependent snRNA regulation, RNA splicing fidelity, and spermatogenic competence, while advocating for biomarker-driven, genotype-tailored therapeutic paradigms in folate-responsive male infertility.

## 1 Introduction

Folic acid (vitamin B9), an essential cofactor in one-carbon metabolism, serves as a critical mediator of nucleic acid biosynthesis and epigenetic regulation, processes indispensable for spermatogenic cell proliferation and genomic stability (1, 2). As a methyl donor in the remethylation of homocysteine to methionine, folic acid facilitates de novo synthesis of purine and thymidylate nucleotides while sustaining DNA methylation patterns–mechanisms vital for chromosomal integrity and meiotic progression during spermatogenesis (2, 3). Clinically, folate deficiency is associated with elevated oxidative stress, uracil misincorporation into DNA, and double-strand break accumulation, collectively compromising spermatozoal viability and function. Emerging evidence highlights folic acid's regulatory role in small nuclear RNA (snRNA)-dependent splicing machinery during germ cell development. Preclinical models demonstrate that folate deprivation disrupts snRNA biogenesis, small nuclear ribonucleoprotein assembly, and spliceosomal fidelity, culminating in aberrant pre-mRNA processing, mitotic spindle checkpoint failures, and meiotic aneuploidy (1, 3, 4). Single-nucleus RNA sequencing (snRNA-seq) has elucidated these perturbations at nucleotide resolution, revealing folate-dependent modulation of snRNA methylation and spliceosome processivity (5, 6). Corroborating these findings, clinical trials and meta-analyses report that folic acid supplementation—particularly in men with MTHFR polymorphisms—enhances seminal parameters (e.g., sperm concentration, progressive motility, and chromatin condensation) (7–9). Nevertheless, interstudy heterogeneity in dosing (0.4–5 mg/day), treatment duration, and concurrent micronutrient administration (e.g., zinc, vitamin B12) yields inconsistent outcomes, complicating evidence-based recommendations (10, 11)

Despite these advances, key knowledge gaps persist. The mechanistic interplay between folate status, snRNA epitranscriptomics, and spliceosome kinetics remains incompletely characterized, with few studies integrating multi-omics (transcriptomic, epigenomic, proteomic) approaches to establish causal pathways (12, 13). Furthermore, unresolved concerns regarding supraphysiologic folate doses potentially inducing transgenerational epigenetic alterations necessitate longitudinal safety assessments and biomarkers for monitoring (10). Addressing these limitations through standardized trial designs, advanced sequencing technologies, and personalized nutrigenomic strategies will be pivotal for translating mechanistic insights into clinical practice.

In this review, we aim to address these gaps by synthesizing current evidence on the molecular and clinical impacts of folic acid in male fertility, with a particular focus on snRNA sequencing and RNA splicing mechanisms. We will (1) elucidate the biological functions of folic acid in one-carbon metabolism and snRNA-mediated splicing during spermatogenesis; (2) examine experimental and clinical data on folic acid deficiency and supplementation; (3) construct molecular regulatory networks integrating snRNA, mRNA, miRNA, and lncRNA data; and (4) critically evaluate limitations and propose precision nutrition strategies, biomarker development, and future research directions to optimize folic acid's therapeutic potential in improving sperm quality.

## 2 Biological role of folic acid in spermatogenesis and snRNA-mediated RNA splicing

### 2.1 Molecular functions of folic acid in one-carbon metabolism and nucleic acid synthesis

Folic acid, an essential B vitamin, plays a significant role in one-carbon metabolism, a pivotal biochemical pathway critical for nucleic acid synthesis. It acts as a methyl donor, aiding the conversion of homocysteine to methionine, thus facilitating the methionine cycle (2). This process is essential for the synthesis of purine and pyrimidine bases, vital components of DNA and RNA (2). Consequently, folic acid supports the rapid cell division and high level of spermatogenesis, ensuring the proliferation and differentiation of germ cells within the seminiferous tubules (3, 14). Its deficiency not only disrupts the methionine cycle but also leads to deoxynucleoside triphosphate (dNTP) pool imbalances, resulting in abnormal DNA metabolism and increased genomic instability, which can have deleterious effects on sperm development (2, 3).

Moreover, folic acid is known to directly influence DNA methylation, which is crucial for maintaining genomic integrity and regulating gene expression. In its absence, uracil misincorporation into DNA can occur, resulting in double-strand breaks and increased mutagenesis rates, further implicating folic acid deficiency in chromosomal instability and impaired spermatogenesis (2). This is corroborated by studies showing altered semen characteristics and a reduction in sperm count in folate-deficient models, emphasizing its role in reproductive health (1, 3). In addition, nutrition is increasingly being recognized as a key factor influencing cell health in multiple tissues, including the male reproductive system, through its regulatory effects on the oxidative-inflammatory pathway. Folate deficiency can induce the accumulation of mitochondrial reactive oxygen species (ROS), leading to sperm DNA damage. A study by Arslan et al. (15) confirmed that nutrition-derived oxidative stress is a core driver of cellular dysfunction, with advanced glycation end products (AGEs) promoting inflammation through RAGE signaling, exacerbating ROS production and cellular dysfunction–a mechanism also associated with male infertility. Folate, acting as a methyl donor and antioxidant, can counteract these effects by supporting redox balance and reducing DNA damage in developing sperm (15).

### 2.2 snRNA architecture and its role in pre-mRNA splicing during spermatogenesis

SnRNAs are critical constituents of the spliceosome, the molecular machinery responsible for removing introns from pre-mRNA, forming mature mRNA transcripts. During spermatogenesis, efficient pre-mRNA splicing is crucial for the proper expression of testis-specific genes (2). The structural configuration of snRNA allows it to form complexes with protein factors, creating small nuclear ribonucleoprotein particles essential for splicing accuracy and efficiency.

The role of snRNA in pre-mRNA splicing during spermatogenesis involves recognizing splice sites on nascent pre-mRNA and catalyzing the removal of introns. This process is fundamental to the precise regulation of gene expression, facilitating the development of spermatozoa with optimal functional capacities. Dysregulation or mutations in snRNA components can impair splicing fidelity, leading to aberrant gene expression and potential defects in spermatozoal structure and function (1, 2).

Emerging evidence suggests that folic acid deficiency perturbs snRNA expression and spliceosome function, leading to dysregulated pre-mRNA splicing in spermatogenic cells (1, 3). Experimental models indicate that folate deficiency exacerbates genomic instability by disrupting RNA splicing mechanisms, leading to defective sperm development and increased rates of apoptosis in germ cells (1, 4).

### 2.3 Impact of snRNA dysregulation on sperm development and quality

Dysregulation of snRNA during RNA splicing, particularly amid folic acid deficiency, directly impacts sperm development and quality. Abnormal snRNA expression leads to splicing errors, resulting in defective mRNA products that impair protein synthesis essential for spermatogenesis (2, 7, 12). This can reduce sperm count and quality by affecting critical attributes such as motility, morphology, and genetic integrity, which are essential for successful fertilization and healthy embryo development.

Clinical studies have demonstrated that folic acid supplementation can mitigate snRNA dysregulation impacts, enhancing semen parameters in men with specific genetic polymorphisms, such as MTHFR mutations (8, 9). Such findings highlight the potential of folic acid in enhancing sperm quality through its regulatory effects on snRNA-mediated RNA splicing.

The mechanistic insights gained from snRNA sequencing highlight the necessity of maintaining adequate folic acid levels to ensure snRNA normal function, thereby supporting optimal spermatozoa formation and preventing reproductive dysfunction (8, 12). As the field advances, these intricate relationships between folic acid, snRNA, and sperm development continue to offer promising avenues for improving male reproductive health through targeted nutritional and therapeutic interventions. Table 1 illustrates molecular functions of folic acid in spermatogenesis and impact on snRNA.

**Table 1:** Molecular functions of folic acid in spermatogenesis and impact on snRNA.

**Molecular function**	**Mechanistic details**	**Impact on snRNA/splicing**	**References**
Methyl donor in one-carbon metabolism	Facilitates homocysteine remethylation to methionine, enabling S-adenosylmethionine (SAM) synthesis for methylation.	Maintains snRNA stability and spliceosome assembly; ensures proper snRNA methylation.	(2, 16)
DNA/RNA synthesis regulation	Provides precursors (purines/pyrimidines) for nucleic acid synthesis during rapid germ cell division.	Supports snRNA transcription; reduces splicing errors by ensuring RNA polymerase II activity.	(1, 18)
Epigenetic regulation	SAM-mediated DNA methylation maintains genomic stability; prevents uracil misincorporation and DNA breaks.	Alters snRNA methylation patterns, affecting spliceosome efficiency and RNA splicing fidelity.	(6, 7)
Oxidative stress mitigation	Scavenges reactive oxygen species (ROS); protects germ cell DNA from oxidative damage.	Prevents oxidative damage to snRNA, preserving spliceosome function and RNA splicing accuracy.	(10, 66)
ER stress modulation	Inhibits PERK pathway activation under folate deficiency, reducing ER stress-induced splicing dysregulation.	Restores snRNA expression and splicing factor availability under folate-deficient conditions.	(12)

## 3 Effects of folic acid deficiency on snRNA expression and RNA splicing: evidence and mechanisms

### 3.1 Folate deficiency-induced genomic instability and spermatogenic impairment

Folic acid deficiency has been increasingly linked to a decline in male reproductive health, particularly through its effects on small nuclear RNA (snRNA) expression and RNA splicing (16). The essential role of folic acid in spermatogenesis is attributed to its involvement in one-carbon metabolism and nucleic acid synthesis, processes critical for germ cell development (17). This section illustrates how folic acid deficiency impacts snRNA expression and RNA splicing mechanisms, providing a comprehensive overview of experimental evidence, associated chromosomal instabilities, and their implications for sperm quality (18).

Numerous studies have employed both animal models and cell culture techniques to investigate the effects of folic acid deficiency on spermatogenesis (18). For instance, studies utilizing rodent models have shown that dietary folate restriction leads to significant reproductive impairments, with resultant decreases in sperm count and motility (19). In controlled experiments, male mice subjected to a low-folate diet exhibited an increase in abnormal sperm morphology and elevated levels of oxidative stress markers in their testes (20). Additionally, ***in vitro*** studies using human sperm cells have revealed that folic acid supplementation correlates with enhanced sperm parameters, such as increased motility and structural integrity, reinforcing the therapeutic potential of folate replenishment (8). These models provide robust mechanisms linking folic acid deficiency to compromised sperm development (9).

At the cellular level, folate deficiency induces meiotic instability through multiple pathways (17). Research has established a connection between reduced folate levels and spindle assembly checkpoint (SAC) dysfunction, which compromises the accurate segregation of chromosomes during meiosis (21). Experiments in murine models have demonstrated that folate deficiency results in mitotic errors, with increased rates of aneuploidy in sperm cells (20). DNA damage response pathways are often activated in these scenarios, suggesting that the lack of adequate folate impacts cell cycle regulation. Such chromosomal instability is associated with increased rates of male subfertility, highlighting the essential role of folic acid in maintaining genomic stability during spermatogenesis (22).

### 3.2 Post-transcriptional and epigenetic disruptions in sperm under folate deficiency

Folic acid's influence extends to the posttranscriptional regulation of gene expression through its impact on snRNA methylation and spliceosome assembly (23). Impaired snRNA methylation has been demonstrated to compromise mRNA splicing fidelity, resulting in the accumulation of aberrantly spliced or unspliced transcripts. *In vivo* studies reveal that folic acid deficiency downregulates key methyltransferases and spliceosomal components, impairing splicing efficiency (19, 20). This disruption in spliceosome functionality results in aberrant splicing events, which can drastically alter the expression profiles of genes pivotal to spermatogenesis. Besides, ***in vitro*** studies have further supported these findings, showing that supplementation with folic acid can restore normal snRNA methylation and splicing efficiency, thereby optimizing sperm quality.

Folic acid deficiency has significant implications on the epigenetic regulation of sperm, impacting the expression of genes involved in germ cell development (21). aberrant histone methylation and dysregulation of DNA methylation have been observed in the context of folate deficiency, highlighting the potential risk for transgenerational effects on offspring (24, 25). Research has demonstrated that low folate levels correlate with altered expression of multiple epigenetic regulators, which can lead to long-lasting modifications in gene expression patterns critical for spermatogenesis (26). Such findings emphasize the necessity of adequate folate intake, not only for current reproductive health but also for preserving the genetic integrity of future generations.

**Figure 1:**
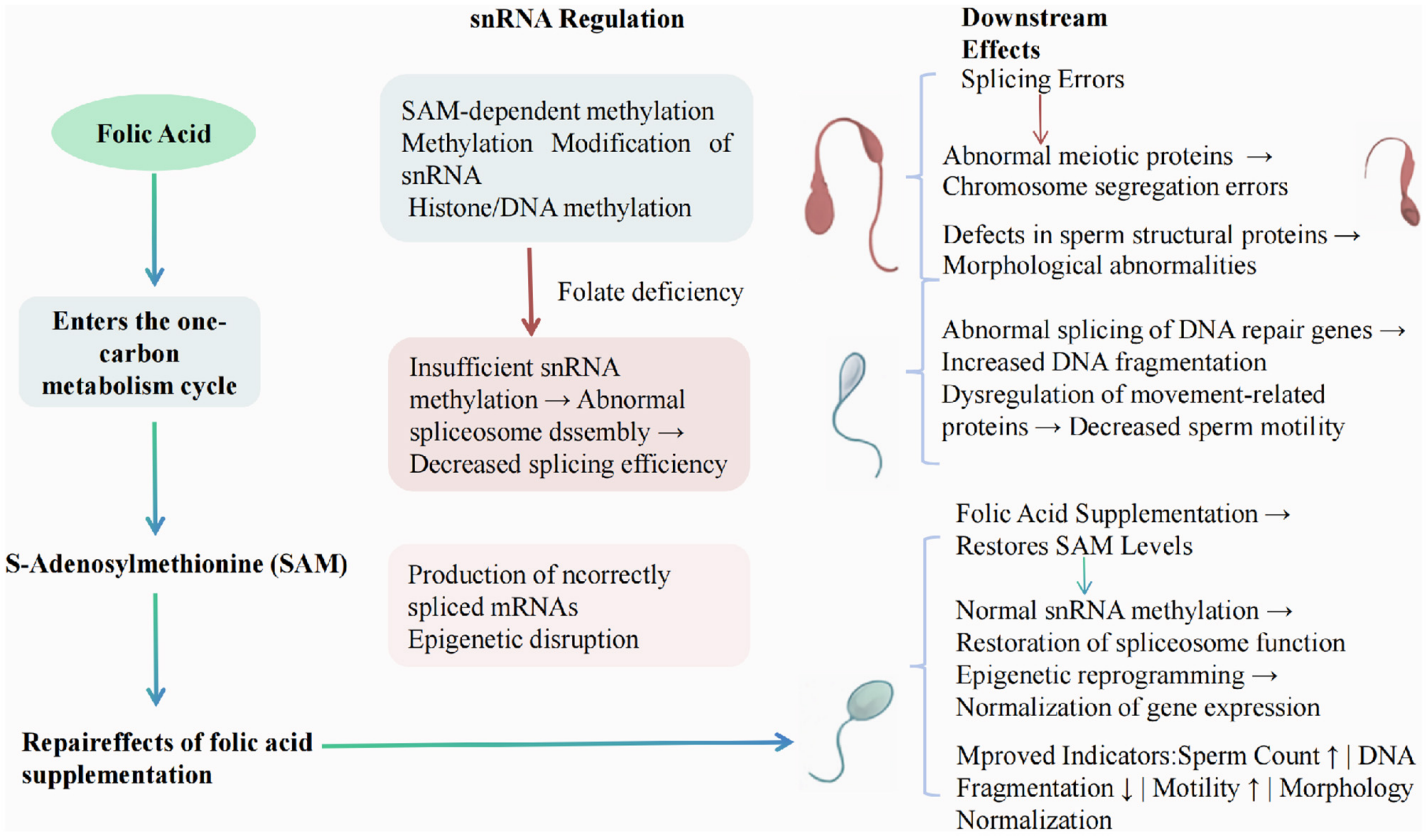
Mechanism of folate deficiency affecting RNA splicing and spermatogenesis through snRNA regulation.

The culmination of disruptions caused by folic acid deficiency results in substantial negative consequences for sperm DNA integrity, morphology, and motility (27). Studies reported that low folate levels lead to increased sperm DNA fragmentation, a critical marker associated with male infertility (28). Furthermore, folic acid deficiency has been linked to morphologically abnormal sperm, characterized by defects in head and tail structures (25, 29). Motility assays have shown that supplementation with folic acid improves both progressive motility and overall sperm vitality, underscoring its role in enhancing key functional parameters of sperm (23). Overall, the evidence reveals that maintaining adequate levels of folic acid is crucial for preserving both the structural and functional attributes of sperm, thereby improving male fertility potential (29). As shown in Figure 1, this is the pathway regulated by snRNA and its relationship with folic acid supplements.

## 4 Clinical and epidemiological evidence for folic acid supplementation in improving sperm quality

### 4.1 Overview of clinical trials on folic acid supplementation and semen parameters

Folic acid supplementation has been the subject of numerous clinical trials aimed at evaluating its effects on semen quality in infertile men. These studies have investigated various parameters, including sperm count, motility, morphology, and DNA integrity (30). Clinical trials have demonstrated improvements in sperm density, particularly in individuals with the MTHFR C677T genotype who received folic acid supplementation, which may play a role in reducing oligoasthenospermia and enhancing reproductive outcomes (8, 9). Additionally, high-dose folic acid supplementation in association with assisted reproductive technologies (ART), such as ***in vitro*** fertilization (IVF) and intracytoplasmic sperm injection (ICSI) has revealed positive effects on fertility metrics, indicating increased biochemical pregnancy rates and decreased sperm DNA fragmentation (30).

Further investigations into the role of folic acid supplementation in reproductive health have extended to exploring the interactions with genetic factors, such as MTHFR polymorphisms and their influence on folate metabolism. Current evidence regarding combination therapies remains inconclusive. While folic acid monotherapy demonstrates modest improvements in progressive motility (10), its combination with zinc fails to show additive benefits on conventional semen parameters (11, 13). These findings underscore the need for precision nutrition strategies in male fertility management.

While numerous studies endorse the beneficial effects of folic acid on sperm quality, there are instances where supplementation has not yielded significant improvements, pointing to the complexity of its interaction with genetic and environmental factors (10). For example, folic acid plus zinc supplementation did not enhance sperm characteristics or lead to better pregnancy outcomes in specific cohorts (13). Additionally, maternal folic acid supplementation during pregnancy did not produce considerable differences in semen parameters in male offspring, further highlighting its variable effects across different populations (31). Similarly, high-dose folic acid supplementation in individuals carrying specific polymorphisms in folate-related enzymes has demonstrated potential heritable DNA methylation defects in sperm, suggesting potential adverse effects on subsequent generations (6, 7). These mixed findings underline the importance of considering individual genetic predispositions and the broader epigenetic context when evaluating folic acid's efficacy in sperm quality enhancement.

### 4.2 Influence of dosage, duration, and individual genetic background

The influence of folic acid supplementation on sperm quality is modulated by factors such as dosage, duration of intervention, and individual genetic background. Specifically, individuals with the MTHFR C677T polymorphism have shown a significant improvement in certain sperm parameters with folic acid supplementation, suggesting that genetic testing could play a pivotal role in tailoring interventions (6, 8). Interestingly, short-term exposure to folic acid supplementation may not lead to notable changes in sperm quality, whereas prolonged exposure might result in altered DNA methylation patterns with significant consequences (6). Determining the optimal dosage and duration requires careful consideration of baseline folate levels, genetic factors, and concurrent nutritional interventions. Studies have indicated that high-dose folic acid supplementation for extended periods might induce DNA methylation defects in sperm, emphasizing the need to balance therapeutic benefits with potential risks (6).

### 4.3 Role of combined supplementation and recommendations for target populations

The role of combined supplementation with folic acid, zinc, and other antioxidants has been extensively explored in clinical contexts, aiming to enhance sperm quality and improve fertility outcomes. While some trials have shown synergistic benefits, leading to improvements in sperm motility and decreased oxidative stress, others have failed to replicate these findings, indicating the need for robust large-scale trials with precise control over variables such as dosage and treatment duration (32, 33). Zinc's involvement in sperm maturation and quality through its effects on sperm membrane stability and motility has made it a target for supplementation strategies; While folic acid supplementation shows promise for improving sperm quality, current evidence presents notable contradictions and limitations that warrant careful consideration. Several well-designed clinical trials, including the large FAZST study, reported null effects on semen parameters and live birth rates despite adequate statistical power (13). Other trials demonstrated biochemical improvements (e.g., reduced oxidative stress) without corresponding functional benefits in sperm motility (32). Moreover, challenges in the methodologies of the studies still exist, with some trials being limited by small sample sizes and others being affected by the lack of blinding, which may introduce bias in subjective assessments. Future research should prioritize large-scale, rigorously controlled trials incorporating standardized protocols, advanced molecular profiling (e.g., snRNA-seq), and long-term safety monitoring to establish evidence-based guidelines. Such efforts must specifically address dose-response relationships, genetic stratification, and the development of predictive biomarkers to enable personalized therapeutic approaches in male fertility management.

Meanwhile, Given the well-documented interindividual variability in therapeutic response, folic acid supplementation strategies should be tailored to specific patient subpopulations with defined genetic predispositions or biochemical evidence of impaired folate metabolism. Additionally, therapies combining folic acid with zinc or antioxidants should be approached cautiously, with considerations for individual metabolic and genetic profiles influencing the therapeutic response (11, 33). Evidence-based guidelines and tailored interventions based on multi-omics data could significantly enhance the efficacy of supplementation strategies, offering a promising path for future investigations in male reproductive health (6, 33). Table 2 summarized current evidence on folic acid supplementation in male fertility.

**Table 2:** Evidence for folic acid supplementation in male fertility.

**Study design**	**Intervention**	**Population**	**Key findings**	**Limitations**	**References**
RCT (MTHFR-C677T)	5 mg/day folic acid for 6 months	Men with MTHFR 677TT genotype	↑ Sperm density (34%), ↓ DNA fragmentation (22%); ↑ live birth rates (18%).	Small sample size (*n* = 120); lacks multiethnic representation.	(8)
Combination trial	Folic acid (5 mg/day) + zinc (15 mg/day)	Idiopathic infertile men	No significant improvement in sperm motility; ↓ oxidative stress markers.	Confounding effects of zinc; variable compliance rates.	(13)
High-dose longitudinal	10 mg/day folic acid for 12 months	Normozoospermic men	↑ Sperm DNA hypermethylation (*p* = 0.003); potential transgenerational epigenetic risks.	No carcinogenicity assessment; limited follow-up on offspring.	(6, 36)
Precision nutrition	Genotype-guided dosing (MTHFR 677CT/TT)	Subfertile men with MTHFR SNPs	↑ Motility (27%) and morphology (15%) in TT carriers; no effect in CC genotype.	High cost of genetic screening; requires validation in larger cohorts.	(9, 54)
Meta-analysis	Folic acid ± antioxidants	15 trials (*n* = 2,450)	Pooled OR for improved motility: 1.45 (95% CI: 1.12–1.88); high heterogeneity.	Variable dosages (0.4–15 mg/day); inconsistent outcome measures.	(10, 64)

## 5 Dose- and time-dependent effects of folic acid on snRNA expression and sperm RNA splicing

### 5.1 Dose-response relationship of folic acid on snRNA levels and RNA splicing events

The dose-response relationship between folic acid supplementation and snRNA levels is a critical aspect of understanding its role in RNA splicing during spermatogenesis. Several studies have illustrated that varied folic acid dosages significantly affect snRNA expression, consequently impacting RNA splicing events. High doses of folic acid have been linked to specific changes in snRNA profiles, suggesting a potential enhancement of splicing activity and possibly improved spermatogenic processes. For instance, research has shown that increased folic acid dosage correlates with elevated snRNA expression, leading to more efficient RNA splicing and better sperm quality (6, 13).

Moreover, studies focusing on snRNA levels post-supplementation reveal a connection between folic acid doses and splicing efficiency, as snRNAs are crucial components of the spliceosome responsible for cutting and joining pre-mRNA segments. Enhanced splicing accuracy, facilitated by adequate snRNA levels, can improve the maturation of sperm cells, highlighting the importance of dosing in supplementation protocols (8, 34). However, it's crucial to consider the potential for both beneficial and adverse effects depending on the folic acid concentration administered (5, 35).

### 5.2 Temporal dynamics of folic acid supplementation and recovery of snRNA profiles

The timing of folic acid supplementation plays a substantial role in the recovery and regulation of snRNA profiles. Experimental evidence points to significant variations in snRNA expression patterns contingent upon the duration of folic acid intake. Short-term supplementation has been associated with modest improvements in snRNA levels and function, whereas longer-term administration often leads to more pronounced and sustainable changes (5, 36).

In a longitudinal study, snRNA profiles in supplemented subjects did not normalize until the end of the intervention period, demonstrating that sustained folic-acid intake is required for full restoration of spliceosomal function (34). Shorter-term trials have reported early shifts in snRNA expression (5) and partial recovery of splicing efficiency (36), but without the prolonged follow-up needed to confirm stable normalization. Careful considerations should be given to the timeframe alongside dosage when designing supplementation regimens to maximize benefits in sperm quality (5, 36).

### 5.3 Molecular mechanisms underlying dose-dependent changes in snRNA methylation and function

The dose-dependent changes in folic acid levels affecting snRNA methylation illuminate molecular mechanisms critical to snRNA function and RNA splicing in sperm cells. Elevated folic acid intake alters snRNA methylation patterns, directly influencing snRNA stability and activity. These modifications may either enhance splicing efficiency through optimized methylation or induce aberrant methylation, leading to dysregulated gene expression (6). The underlying mechanism involves folic acid's contribution to one-carbon metabolism, which supports methyl group availability for RNA modification. Increased folic acid supplementation can augment snRNA methylation, thereby improving spliceosome assembly fidelity (35, 37). However, supraphysiological doses may disrupt methylation homeostasis, underscoring the importance of precise dosage calibration (6).

A comprehensive understanding of these molecular mechanisms will facilitate the crafting of supplementation strategies tailored to individual genetic and physiological contexts (6, 37).

### 5.4 Implications for optimizing supplementation regimens

The implications of varying doses and supplementation durations for folic acid are profound, necessitating personalized regimens to optimize sperm quality and RNA splicing efficiency. Insights garnered from dose-dependent studies suggest that an optimal supplementation regimen must account for individual variability in genetic predispositions and current health status, particularly considering polymorphisms like MTHFR which affect folic acid metabolism (8, 9).

**Figure 2:**
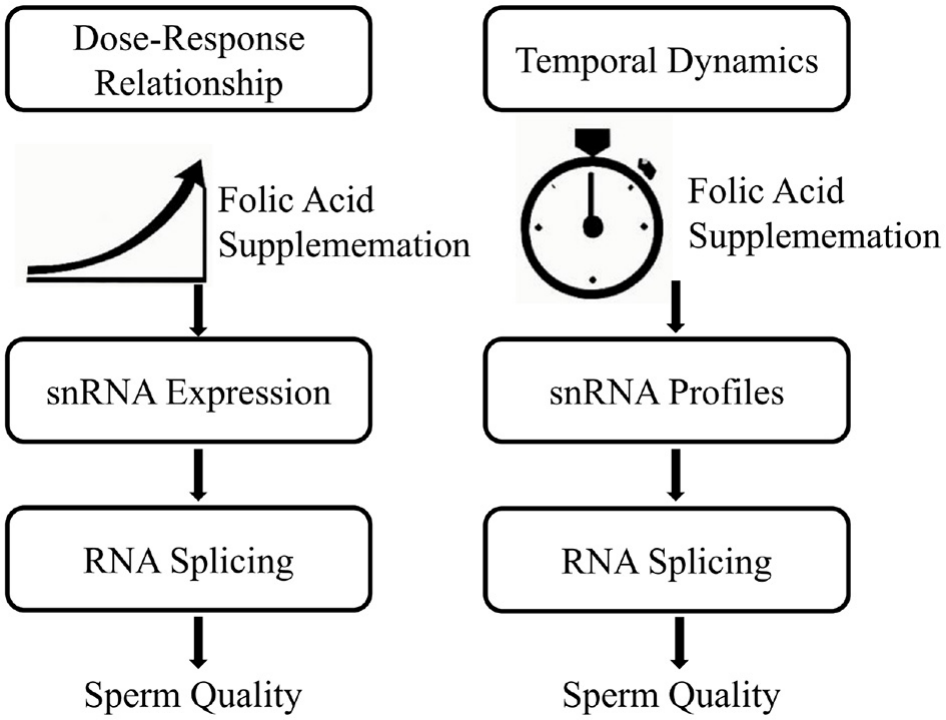
Dose-time response relationship of folic acid on snRNA expression and RNA splicing events.

Establishing suitable dosage and timing protocols not only enhances snRNA functionality and sperm quality but also reduces potential risks associated with excessive folic acid intake or prolonged supplementation (9, 38). It is recommended that clinicians adopt a patient-specific approach, incorporating genetic screenings to tailor dosages effectively and minimize adverse effects. Furthermore, regular monitoring of sperm RNA splicing parameters can provide feedback to refine regimens, ensuring the most beneficial outcomes for male reproductive health (13, 38). Figure 2 is a dose-time response relationship diagram of folic acid on snRNA expression and RNA splicing events.

Overall, the integration of comprehensive biological, molecular, and clinical data will be pivotal in guiding future research and clinical applications of folic acid supplementation in enhancing male fertility (9, 39).

## 6 Construction of molecular regulatory networks via snRNA sequencing to elucidate folic acid's role

### 6.1 Application of snRNA sequencing to identify differentially expressed snRNAs after folic acid intervention

The utilization of snRNA-seq provides an innovative avenue for identifying differentially expressed snRNAs following folic acid intervention, which is critical for comprehending its effects on sperm RNA splicing and quality. By isolating single nuclei from spermatogenic cells, researchers can map the expression patterns of snRNAs before and after folic acid supplementation, thus highlighting the specific snRNA alterations attributable to this vitamin. A recent study used this approach to explore the cellular heterogeneity in diseased tissues, suggesting its applicability in reproductive health (40).

SnRNA-seq allows for the identification of unique clusters of snRNAs that might be up- or down-regulated in response to folic acid. For instance, in experimental models, folic acid supplementation can lead to distinct transcriptional changes in snRNA profiles, implicating these RNAs in RNA splicing fidelity and efficiency. This high-resolution technique has the potential to precisely delineate how folic acid modulates the splicing machinery in spermatogenic cells, aligning with exist findings that differential splicing correlates with improved sperm function under adequate folic acid levels (41, 42).

### 6.2 Integration of mRNA, miRNA, and lncRNA data for comprehensive network modeling

The construction of a comprehensive molecular regulatory network necessitates the systematic integration of diverse RNA species, encompassing mRNAs, miRNAs, and lncRNAs. Such integration can illuminate the complex interactions and pathways modulated by folic acid. For instance, when applied to the study of cognitive impairments, combined lncRNA and mRNA datasets have provided insights into potential therapeutic pathways, underscoring the value of multi-omics data in network modeling (26). Regarding male fertility studies, establishing a robust regulatory network requires precise correlation of snRNA modifications with their downstream mRNA targets and associated miRNA regulators. Such a systems-level perspective enables a more comprehensive understanding of folic acid's cascading effects on gene expression regulation and RNA splicing dynamics. Pathway-centric correlation analyses offer particularly valuable insights into the interconnected biological processes governing spermatogenesis, including DNA replication/repair mechanisms, signal transduction cascades, and metabolic regulation (43).

Identifying key regulatory nodes and pathways modulated by folic acid constitutes a pivotal step in understanding its biological role. This involves recognizing which transcription factors, RNA-binding proteins, and other regulatory elements are impacted by folic acid-mediated snRNA changes. Noteworthy nodes often include master transcription factors or miRNAs that orchestrate large-scale physiological responses or cellular adaptations (44, 45).

For example, the integration of snRNA data with hub genes in folate biosynthesis or metabolism pathways could help identify regulatory nodes critical for maintaining genomic stability during spermatogenesis. Such findings can be leveraged to explore therapeutic interventions aimed at mitigating folate-deficiency-related anomalies by stabilizing these nodes. Moreover, computational tools such as FFLatt can be used to generate realistic gene regulatory networks. In the context of folate's impact on sperm quality, FFLatt can identify key folate-affected gene regulatory feedforward loops by analyzing snRNA sequencing, mRNA, and miRNA expression data. It can also simulate the dynamic behavior of these feedforward loops under different folate concentrations or treatment conditions. By conducting functional enrichment analysis on the identified FFLs, the specific roles of these regulatory modules in biological processes such as sperm development, motility, and DNA integrity can be inferred. This provides predictive insights into network dynamics and interactions between nodes, which is crucial for understanding the mechanisms of folate action (44, 46).

The specific modeling process of FFLatt can construct miRNA-mRNA regulatory pairs based on the target prediction algorithms of miRNA and mRNA and their negatively correlated expression data. lncRNA can competitively bind to miRNA by acting as a “sponge" for miRNA, thereby relieving the inhibition of miRNA on mRNA and forming a complex ceRNA network. Given the central role of snRNA in the RNA splicing mechanism, its abnormalities may lead to changes in mRNA splicing patterns. By analyzing differential splicing events, the impact of specific snRNAs on mRNA splicing products can be inferred. The constructed gene regulatory network is input into the FFLatt tool to identify feedforward loop structures. In the constructed network, highly connected hub genes or RNA molecules are identified, which may be the core regulators through which folate exerts its effects. GO and KEGG pathway enrichment analyses are performed on differentially expressed genes and key regulatory nodes in the network to determine the specific biological processes and signaling pathways through which folate affects sperm quality (47, 48).

### 6.3 Validation of network-based mechanisms linking folic acid, snRNA, and sperm quality

**Figure 3:**
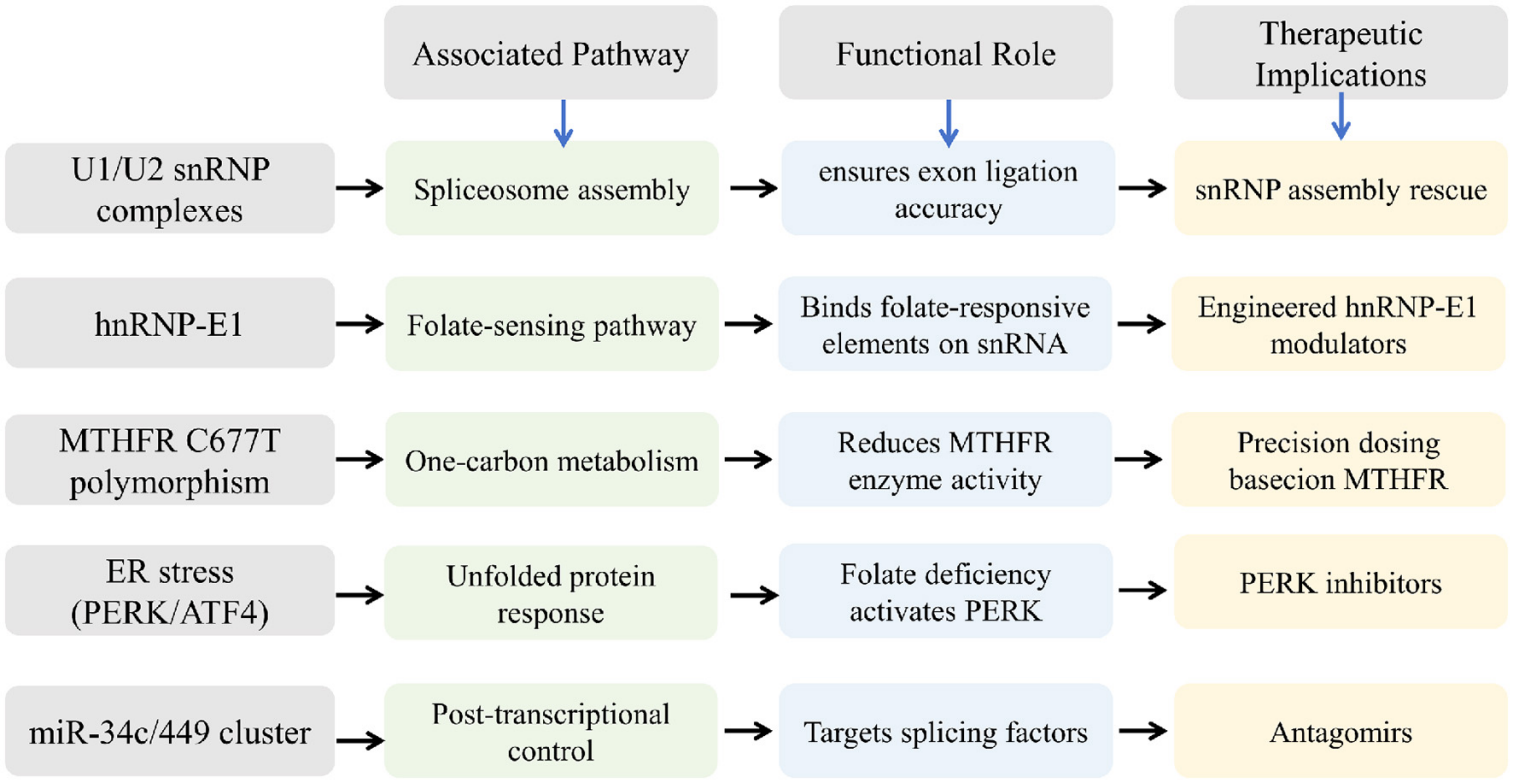
Possible regulatory networks and pathways identified by snRNA sequencing.

The validation of the proposed regulatory networks is crucial to ensure their reliability and applicability in clinical scenarios. Techniques such as quantitative Polymerase Chain Reaction (qPCR), RNA interference, and chromatin immunoprecipitation aid in experimentally verifying the interactions and effects predicted by network models. These methodologies can corroborate the roles of specific snRNAs in enhancing sperm function under folic acid influence (49). Figure 3 shows the possible regulatory networks and pathways identified by snoRNA sequencing.

Furthermore, evaluating the impacts of folic acid through a combination of genomics and phenotypic assessments allows for a clearer picture of its role in sperm quality. For example, high-throughput interactome screening has uncovered potential RNA-protein networks where RNA fragments play parts, offering insights into RNA-mediated regulatory mechanisms (50). Such findings are particularly valuable for translating these molecular insights into actionable therapeutic strategies.

Overall, constructing molecular regulatory networks using snRNA sequencing and integrating multi-omics data offers a promising pathway to elucidate the multifaceted role of folic acid in reproductive health. These efforts are critical in advancing personalized nutritional strategies and therapeutic interventions, ultimately enhancing male fertility. This network-based approach highlights the interconnectedness of molecular processes regulated by folic acid, underscoring its importance in maintaining sperm quality and functionality through precise regulatory mechanisms (51, 52).

## 7 Controversies in the molecular mechanisms of folic acid and breakthroughs in multi-omics research

### 7.1 Comparison of competing hypotheses regarding folic acid's molecular targets

The role of folic acid in male fertility has been examined through various hypotheses, primarily focusing on its molecular targets. One prevailing hypothesis is that folic acid directly influences sperm count and quality by impacting DNA methylation patterns in sperm cells. This hypothesis is strongly supported by studies showing that folic acid supplementation leads to a decrease in sperm DNA fragmentation and improves seminal parameters, particularly in those with the MTHFR 677 TT genotype (8, 9, 11). Another hypothesis suggests that folic acid modulates the expression of key genes involved in spermatogenesis, highlighting the importance of folic acid in cellular pathways related to sperm development (8, 30).

Alternatively, some studies posit that folic acid's role may extend beyond direct genomic effects. For instance, folic acid deficiency has been shown to influence the endoplasmic reticulum (ER) stress pathway, thereby affecting overall cellular health and fertility (12). There is also a hypothesis that folic acid interacts with zinc and other antioxidants, creating synergies that enhance sperm quality and increase live birth rates among couples undergoing fertility treatment (11, 13). However, conflicting evidence from various trials casts doubt on the consistency of these benefits, necessitating further investigation.

### 7.2 Research gaps and multi-omics strategies

Despite significant advancements, there exist notable limitations and gaps in the current body of research concerning folic acid's role in male fertility (see Table 3). Clinical studies often struggle with heterogeneity in sample populations and inconsistencies in supplementation protocols, which complicate the interpretation of results. Differences in dosages, duration, and individual genetic backgrounds pose challenges in forming conclusive statements regarding the efficacy of folic acid supplementation (10, 33, 53).

**Table 3:** Research gaps and future directions.

**Critical gap**	**Proposed solution**	**Expected outcome**
Inconsistent clinical outcomes	Stratified RCTs based on MTHFR genotypes, epigenetic profiles, and baseline folate.	Personalized supplementation protocols with higher efficacy.
Unknown long-term safety	Cohort studies tracking offspring of men receiving high-dose folate (>5 mg/day).	Clarify transgenerational epigenetic risks (e.g., DNA hypermethylation).
Mechanistic ambiguity in snRNA-sperm link	Multi-omics integration (snRNA-seq, methylome, proteome) in human spermatogonia.	Map dynamic folate-responsive networks driving splicing fidelity.
Poor synergy with co-supplements	Dose-response surface modeling for folate + zinc/antioxidants.	Optimized combination regimens for maximal sperm quality improvement.
Lack of real-time monitoring tools	Develop CRISPR-based snRNA reporters or nanopore sequencing for live sperm analysis.	Enable dynamic assessment of splicing efficiency during treatment.

Although folic acid's role in DNA methylation and spermatogenesis has been extensively characterized, the precise molecular mechanisms and regulatory pathways remain incompletely elucidated. Many studies do not explore the mechanistic underpinnings in-depth, leaving gaps in our understanding of how folic acid mediates its effects at the molecular level (12, 13). Moreover, integrated multi-omics analyses incorporating snRNA profiling, transcriptomic data, and epigenetic modifications remain limited, despite their potential to unravel the complex, multifactorial impact of folic acid on male reproductive function.

To address the existing gaps, an integration of multi-omics data represents a promising avenue for mechanistic clarification. By combining snRNA sequencing with mRNA, miRNA, and lncRNA profiles, researchers can construct comprehensive molecular regulatory networks that define folic acid's role in sperm development. Such an integrated approach allows for the identification of key regulatory nodes and pathways, providing insights into complex interactions between genetic and environmental factors impacting fertility (9).

Integrating omics data can also facilitate the identification of biomarkers. By bridging clinical findings with robust mechanistic data, researchers can propose targeted interventions, potentially transforming folic acid supplementation in fertility treatments (7, 54). The incorporation of comprehensive data analysis tools and network modeling approaches thus paves the way for high-resolution mapping of folic acid's impact on male reproductive health. This perspective holds promise for the development of precise, effective supplementation regimens and underscores the essential role of integrative research in clarifying the complex biological functions of folic acid (55).

## 8 Perspectives and future directions in research and clinical practice

### 8.1 Precision nutritional strategies tailored to genetic and epigenetic profiles

As we advance our understanding of the molecular mechanisms underlying male fertility, the opportunity to develop precision nutritional strategies becomes increasingly vital. Folic acid, a pivotal factor in one-carbon metabolism, exhibits differential effects on sperm quality depending on individual genetic variations, such as the common MTHFR C677T polymorphism (8, 9). This polymorphism can lead to elevated homocysteine levels and increased DNA damage, thereby necessitating individualized supplementation protocols to optimize sperm health. Precision nutrition involves tailoring dietary interventions based on genetic predispositions and epigenetic modifications, potentially enhancing outcomes in individuals with specific genetic markers.

Epigenetic profiling, specifically DNA methylation patterns, may further guide personalized nutrition strategies. Research has shown that folic acid supplementation can modify sperm methylation profiles, impacting germ cell function and inheritance across generations (5, 56). By integrating genetic and epigenetic data, healthcare providers can offer customized recommendations, potentially improving reproductive outcomes and reducing fertility-related issues.

### 8.2 Development of biomarkers based on snRNA expression and RNA splicing patterns

Biomarkers serve as valuable tools in diagnosing and monitoring various health conditions. Emerging evidence suggests that snRNA expression levels and splicing patterns are promising biomarkers for assessing sperm quality and identifying folic acid-responsive changes (55, 57). Given their regulatory roles in spermatogenic processes through RNA splicing modulation, these molecular signatures could provide valuable indicators of male fertility status and therapeutic response.

Investigations of snRNA-centric regulatory networks, particularly their interactions with other non-coding RNA species, may reveal novel biomarkers associated with male reproductive dysfunction. These molecular indicators could prove critical for detecting folic acid deficiency-induced splicing abnormalities and informing targeted treatment strategies. The implementation of high-throughput genomic technologies, notably RNA sequencing approaches, will be essential for biomarker validation and clinical translation (58, 59).

### 8.3 Potential therapeutic interventions targeting snRNA processing and methylation

The intricate role of snRNA in RNA splicing and gene regulation highlights its therapeutic potential. Modulating snRNA processing and methylation could emerge as a novel strategy in addressing male infertility issues related to folic acid deficiency (3, 7). Therapeutic interventions could target specific RNA-binding proteins or small RNAs involved in these processes, thereby restoring normal cellular function and enhancing spermatogenesis.

Effective therapies might encompass folic acid supplementation regimes adjusted to modify RNA methylation patterns and snRNA activity. Additionally, drugs or supplements that specifically target the molecular machinery of RNA splicing could offer new avenues for treatment. The development of such interventions requires rigorous research to elucidate the precise mechanisms and optimize strategies for clinical application.

### 8.4 Emerging technologies for real-time monitoring of RNA splicing and sperm quality

Technological advancements continue to revolutionize our ability to study biological processes in real-time. Innovative technologies like single-molecule RNA sequencing and live-cell imaging offer new possibilities for monitoring RNA splicing and sperm quality dynamics. These tools enable researchers to visualize and quantify splicing events, snRNA activity, and other key cellular processes that influence fertility in a high-throughput precise manner. Real-time monitoring can facilitate the identification of immediate changes resulting from nutritional or therapeutic interventions, allowing clinicians to adjust treatment plans promptly. For instance, observing alterations in sperm RNA profiles following folic acid supplementation can provide direct feedback on intervention effectiveness. As these technologies evolve, they will likely become integral components of fertility assessments and treatment strategies, driving personalized medicine forward.

Emerging technologies such as single-molecule RNA sequencing and live-cell imaging now enable real-time monitoring of RNA splicing dynamics and sperm quality, providing high-resolution quantification of snRNA activity, spliceosome efficiency, and transcriptomic alterations in spermatozoa (60).

These advancements offer unprecedented insights into folate-responsive splicing fidelity. For instance, Nanopore sequencing can detect aberrant splicing isoforms in sperm RNA within 24–48 h, serving as a potential diagnostic tool for folate-mediated splicing defects in male infertility (61). CRISPR-based reporters may be used to visually track splicing accuracy in live sperm cells, but clinical application may require 3-5 years for validation and standardization. In the short term, clinicians may adopt periodic assessments of sperm RNA splicing profiles (e.g., RT-qPCR of key splice variants) to adjust folate supplementation regimens based on splicing efficiency biomarkers, whereas point-of-care microfluidics could eventually provide rapid splicing readouts despite current cost and technical limitations. Over the long term, simplified assays such as methylation-sensitive splicing panels are expected to become routine in andrology labs, particularly for men with MTHFR variants or idiopathic infertility. To bridge the gap between research and clinical implementation, future efforts must prioritize the validation of splicing biomarkers in multicenter trials and optimize the cost-effectiveness of real-time platforms. Collectively, these innovations promise to transform the management of folate-responsive male infertility through precision diagnostics and tailored therapeutic interventions.

## 9 Conclusions

Folic acid plays a pivotal role in male reproductive health, primarily through its influence on snRNA-mediated RNA splicing and one-carbon metabolism, which are essential for maintaining genomic stability and spermatogenesis (62). This review synthesizes current evidence to highlight the multifaceted mechanisms by which folic acid deficiency disrupts sperm quality, including impaired snRNA maturation, aberrant DNA methylation, and increased oxidative stress. Conversely, supplementation has shown promise in improving seminal parameters, particularly in individuals with genetic predispositions such as MTHFR polymorphisms (63, 64). However, the efficacy of folic acid is highly context-dependent, influenced by factors such as dosage, duration, and interactions with other micronutrients.

Despite these advances, critical gaps remain. The precise molecular pathways linking folic acid to snRNA regulation and splicing fidelity require further elucidation, particularly through multi-omics approaches that integrate epigenomic, transcriptomic, and proteomic data. Additionally, the long-term safety of high-dose supplementation and its potential transgenerational epigenetic effects necessitate rigorous investigation (65, 66). Future research should prioritize stratified clinical trials, biomarker development, and real-time monitoring technologies to optimize personalized supplementation strategies (67, 68).

In summary, while folic acid represents a promising intervention for male infertility, its application must be guided by precision medicine principles, combining genetic profiling, advanced omics technologies, and tailored therapeutic regimens (69). Addressing these challenges will not only enhance our understanding of folic acid's role in reproductive health but also pave the way for more effective and safer interventions in clinical practice.
